# Contact sensitization to hydroperoxides of limonene and linalool: Results of consecutive patch testing and clinical relevance

**DOI:** 10.1111/cod.13137

**Published:** 2018-10-31

**Authors:** Daan Dittmar, Marie L. A. Schuttelaar

**Affiliations:** ^1^ Department of Dermatology University of Groningen, University Medical Centre Groningen Groningen The Netherlands

**Keywords:** allergic contact dermatitis, clinical relevance, contact allergy, fragrances, hydroperoxides of limonene, hydroperoxides of linalool, patch testing

## Abstract

**Background:**

Hydroperoxides of limonene and linalool are potent sensitizers.

**Objectives:**

To investigate the prevalence of contact allergy to both hydroperoxides of limonene and hydroperoxides of linalool, to report clinical relevance, and to investigate patient demographics.

**Methods:**

A total of 821 patients (35.6% male, mean age 42.4 years ± 17.8 years) were consecutively patch tested with our departmental baseline series and our fragrance series, including hydroperoxides of limonene 0.3% pet. and hydroperoxides of linalool 1.0% pet. The clinical relevance was assessed for all positive reactions.

**Results:**

Positive patch test reactions to hydroperoxides of limonene and to hydroperoxides of linalool were observed in 77 patients (9.4%, 95% confidence interval [CI]: 7.4%‐11.4%) and in 96 patients (11.7%, 95%CI: 9.5%‐13.9%), respectively; 38 of these patients (4.6%, 95%CI: 3.2%‐6.0%) reacted to both. Most reactions were considered to be possibly or probably clinically relevant (66.3% and 68.8%, respectively), and a small proportion were deemed to be of certain clinical relevance (18.2% and 19.8%, respectively).

**Conclusion:**

As compared with previous studies, high numbers of positive reactions to both hydroperoxides of limonene and hydroperoxides of linalool were observed, the majority of which were clinically relevant, supporting their inclusion in the European baseline series.

## Graphical abstract

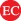

## 1 INTRODUCTION

Limonene (d‐limonene) is the main ingredient of pressed oil from the peel of citrus fruits, and linalool is present in many herbs, flowers, woods, etc.^1^, ^2^ Both are common ingredients in household products and cosmetics, such as hygiene products, perfumes, and detergents, as well as industrial products.^3^, ^4^ Limonene and linalool are ubiquitous fragrance terpenes with low sensitizing potential.^1^, ^2^, ^5^ However, upon air exposure, oxidation occurs, during which different oxidation products are formed. Of these oxidation products, the hydroperoxides are potent sensitizers. High prevalences of contact allergy to these hydroperoxides of limonene and linalool have been reported.^6^, ^7^, ^8^, ^9^ The aim of the current study was to investigate the prevalences of contact allergy to hydroperoxides of limonene and hydroperoxides of linalool, and to characterize patients allergic to either or both hydroperoxides of limonene and hydroperoxides of linalool, with respect to patient characteristics and concomitant fragrance contact allergies.

## 2 METHODS

A database study was performed on all patients who were at least patch tested with both hydroperoxides of limonene 0.3% pet. and hydroperoxides of linalool 1.0% pet. All patients referred to our tertiary referral centre with suspected allergic contact dermatitis (ACD) are consecutively patch tested with our departmental extended European baseline series (EBS), TRUE Test panels 1 and 2 (SmartPractice Europe, Reinbek, Germany) supplemented with additional investigator‐loaded allergens, and a fragrance series. When specific contact allergies are suspected, additional series are patch tested. All investigator‐loaded allergens were tested in Van der Bend square chambers (Van der Bend, Brielle, The Netherlands), and all patch tests were attached to the back with Fixomull stretch (BSN Medical, Hamburg, Germany) for 2 days. Both hydroperoxides of limonene 0.3% pet. and hydroperoxides of linalool 1.0% pet. (Chemotechnique Diagnostics, Vellinge, Sweden) were included in our fragrance series from December 1, 2015.

Consecutively patch tested dermatitis patients from December 1, 2015 to December 15, 2017 were included in the current analysis. All patch tests were read and interpreted by the same dermatologist, with potential back‐up from a dermatologist also trained in reading and interpreting patch test results, according to ICDRG/ESCD criteria, with the possible outcomes being: negative, irritant, doubtful (?+), weak positive (+), strong positive (++), and extreme positive (+++) reactions.^10^ Reactions were considered to be irritant if margins were sharply demarcated and the surface of the test area showed a silk paper structure or a shiny skin. Reactions were considered to be doubtful if erythema and infiltration did not cover the whole test area. Readings were performed on day (D) 3 and D7. For the present analysis, the maximum patch test reactions of these 2 readings were aggregated as the patch test outcome. The distribution of the strength of positive patch test reactions to the hydroperoxides are presented for different groups of patients: patients with positive reactions to either hydroperoxides of limonene or hydroperoxides of linalool but not to any other fragrance; patients with positive reactions to both hydroperoxides of limonene and hydroperoxides of linalool but not to any other fragrance; patients with positive reactions to hydroperoxides of limonene or hydroperoxides of linalool and to ≥1 other fragrances; and patients with positive reactions to both hydroperoxides of limonene and hydroperoxides of linalool and to ≥1 other fragrances.

### 2.1 Clinical relevance and additional contact allergies

For all positive patch test reactions, the current and/or past clinical relevance was determined based on patient history and exposure, with possible outcomes being unlikely/not, possible, probable, and certain. “Unlikely/not” suggested that there was no suspected ACD, “possible” suggested that there was some suspicion of a relationship between the allergen and the dermatitis (between 1% and 49% convinced), “probable” suggested that this suspicion was stronger (between 50% and 99% convinced), and “certain” meant that the relationship was proven (100% convinced) by the presence of allergen in a product to which there was exposure at the body site where there was dermatitis, with a clear temporal relationship. For hydroperoxides of limonene and hydroperoxides of linalool, patients were instructed to review the labelling of their products for either limonene (or d‐limonene, also known as *R*‐limonene, and its enantiomer *S*‐limonene), or linalool, respectively, as ingredients in their products. Their findings were subsequently discussed at our outpatient clinic; if patients were unsure or unable to review their products, they were instructed to bring all of their suspected products for review by the dermatologist. Clinical relevance is presented for the same groups as described above for strength of patch test reaction. The types of product for which exposure caused ACD in patients with a contact allergy to hydroperoxides of limonene and/or hydroperoxides of linalool of certain clinical relevance are presented.

To evaluate concomitant reactions in patients with contact allergy to hydroperoxides of limonene, hydroperoxides of linalool, or both, the proportion of patients with at least ≥1 additional contact allergies apart from allergy to either hydroperoxides of limonene or hydroperoxides of linalool, and the proportion of patients with at least ≥1 additional non‐fragrance allergies (excluding colophonium), are presented. Additional contact allergies were not limited to EBS allergens; that is, any contact allergy was considered.

### 2.2 Data analysis

**Figure 1:**
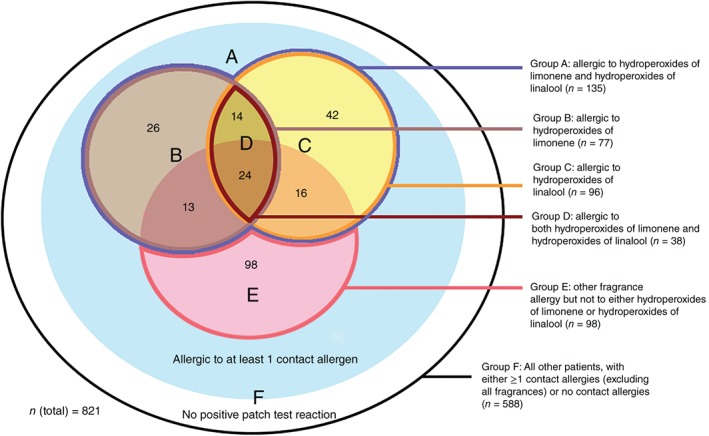
Venn diagram illustrating the different groups of patients based on their patch test outcomes

For data analysis, different groups of patients were defined (Figure 1). Group A comprised all patients with at least positive patch test reactions to hydroperoxides of linonene and/or hydroperoxides of linalool. Group B and group C comprised all patients with positive patch test reactions to hydroperoxides of limonene and hydroperoxides of linalool, respectively. Group D comprised all patients with positive patch test reactions to both hydroperoxides of limonene and hydroperoxides of linalool. In other words, groups B‐D are subsets of group A. Group E comprised all patients with at least 1 positive patch test reaction to a patch tested fragrance allergen but not to hydroperoxides of limonene/linalool, and group F comprised all other patch tested patients. Table S1 provides an overview of all fragrance allergens tested, including the tested concentration and vehicle, in the current cohort of patients. Patient demographics for these groups were described according to the MOAHLFA index.^11^

### 2.3 Statistics

Statistical analyses were performed according to pertinent guidelines.^12^ Prevalences are provided in percentages together with 95% confidence intervals (CIs). The reaction index (RI) (showing the proportion of doubtful/irritant reactions relative to positive reactions, calculated with the formula [positive reactions – (doubtful reactions + irritant reactions)]/[positive + doubtful + irritant reactions], giving a value between −1 and 1) and positivity ratio (PR) (proportion of weak positive [+] reactions among all positive reactions) were calculated for both hydroperoxides of limonene and hydroperoxides of linalool.^13^, ^14^ For variables with a normal distribution, the mean and SD are given; for non‐normally distributed variables, median and interquartile range are given. Logistic regression analysis was performed to assess the risk factors for being in 1 of the above‐mentioned groups as compared with not being in that specific group. Both univariable and multivariable regression analyses were performed; for the multivariable regression analysis, all variables which reached a *P*‐value of <0.1 in the univariable analysis were included. Variables analysed were: sex, age ≥ 40 years, (a history of) atopic dermatitis, occupational dermatitis, and primary site of dermatitis (generalized, trunk, hand, face, leg, and other). All *P*‐values of <0.05 were regarded as being statistically significant. Statistical analyses were performed with spss v.23 (IBM) and Excel 2013 (Microsoft).

## 3 RESULTS

### 3.1 Hydroperoxides of limonene and linalool

A total of 821 patients (35.6% male, mean age 42.4 years ± 17.8 years) were tested with at least our departmental baseline series and our fragrance series including hydroperoxides of limonene and hydroperoxides of linalool. Altogether 77 patients (9.4%, 95%CI: 7.4%‐11.4%) had positive patch test reactions to hydroperoxides of limonene, and 96 patients (11.7%, 95%CI: 9.5%‐13.9%) had positive patch test reactions to hydroperoxides of linalool. The overlap between these 2 groups was 38 patients (4.6%, 95%CI: 3.2%‐6.0%) who had positive reactions to both hydroperoxides of limonene and hydroperoxides of linalool. An overview of the patch test reactions to both hydroperoxides of limonene and hydroperoxides of linalool is shown in Table 1. A total of 141 doubtful (?+) reactions (17.2%, 95%CI: 14.6%‐19.7%) and 7 irritant reactions (0.9%, 95%CI: 0.2%‐1.5%) to hydroperoxides of limonene were observed, and 180 doubtful reactions (21.9% 95%CI: 19.1%‐24.8%) and 16 irritant reactions (1.9%, 95%CI: 1.1%‐2.8%) to hydroperoxides of linalool were observed. The RI and PR for hydroperoxides of limonene were −0.32 (95%CI: −0.41 to −0.24) and 85.7% (95%CI: 77.9%‐93.5%), respectively, and the RI and PR for hydroperoxides of linalool were − 0.34 (95%CI: −0.42 to −0.26) and 88.5% (95%CI: 79.2%‐94.9%), respectively. Of all positive reactions to hydroperoxides of limonene, 6 (7.8%) were either negative or doubtful on the D3 reading, and became positive on D7; for hydroperoxides of linalool, this was seen in 4 cases (4.2%). For both d‐limonene 2.0% pet. and linalool 10.0% pet., 1 positive reaction per allergen was observed in 2 different patients, both of whom were also allergic to both hydroperoxides of limonene and hydroperoxides of linalool.

**Table 1:** Cross table of all patch test reactions (*n* = 821) to hydroperoxides of limonene 0.3% pet. and hydroperoxides of linalool 1.0% pet

	Hydroperoxides of linalool						
	Negative	Irritant	?+	+	++	+++	Total
Hydroperoxides of limonene							
Negative	479	10	70	37	0	0	596
Irritant	2	4	1	0	0	0	7
?+	37	2	81	19	2	0	141
+	9	0	26	26	5	0	66
++	2	0	2	3	3	1	11
+++	0	0	0	0	0	0	0
Total	529	16	180	85	10	1	821

### 3.2 Strength of reactions

As can be deduced from the calculated PRs shown above, the majority of positive reactions to both hydroperoxides of limonene and hydroperoxides of linalool were weak (+) positive. Only 1 extreme (+++) positive reaction to hydroperoxides of linalool was observed. Table 2 shows the distribution of the strength of reactions for the different groups of patients. It can be observed that patients who reacted to only hydroperoxides of limonene or hydroperoxides of linalool had only weak (+) positive reactions, whereas patients who reacted to both hydroperoxides of limonene and hydroperoxides of linalool and at least 1 additional fragrance had a higher proportion of strong (++) positive reactions to hydroperoxides of limonene and/or hydroperoxides of linalool.

**Table 2:** The distribution of the varying degrees of positive reactions to both hydroperoxides of limonene and hydroperoxides of linalool, presented for different groups of patients; patients with positive reactions to hydroperoxides of limonene or hydroperoxides of linalool, but not to any other fragrance; patients with positive reactions to both hydroperoxides of limonene and hydroperoxides of linalool, but not to any other fragrance; patients with positive reactions to hydroperoxides of limonene or hydroperoxides of linalool, and to ≥1 other fragrances; and patients with positive reactions to both hydroperoxides of limonene and hydroperoxides of linalool, and to ≥1 other fragrances

			Strength of patch test reaction, *n* (%)		
Reaction profile		*N* (total)	Weak (+)	Strong (++)	Extreme (+++)
Only hydroperoxides of limonene	Limonene	26	26 (100.0)	0	0
Only hydroperoxides of linalool	Linalool	42	42 (100.0)	0	0
Both hydroperoxides of limolene and hydroperoxides of linalool	Limonene	16	15 (93.8)	1 (6.3)	0
	Linalool	16	13 (81.3)	3 (18.8)	0
Hydroperoxides of limonene + other fragrance	Limonene	13	9 (69.2)	4 (30.8)	0
Hydroperoxides of linalool + other fragrance	Linalool	16	14 (87.5)	2 (12.5)	0
Both hydroperoxides of limonene and hydroperoxides of linalool + other fragrance	Limonene	22	16 (72.7)	6 (27.3)	0
	Linalool	22	16 (72.2)	5 (22.7)	1 (4.5)
Total hydroperoxides of limonene	Limonene	77	66 (85.7)	11 (14.3)	0
Total hydroperoxides of linalool	Linalool	96	85 (88.5)	10 (10.4)	1 (1.0)

### 3.3 Clinical relevance

The clinical relevance of the positive reactions to both hydroperoxides of limonene and hydroperoxides of linalool were evaluated according to the groups described above (Table 3). Positive reactions to hydroperoxides of limonene/hydroperoxides of linalool in patients who were allergic to both, with or without additional fragrance allergies, were more frequently of certain clinical relevance (ranging from 27.3% to 37.5%, not statistically significant). The majority of reactions were evaluated as being of possible or probable clinical relevance. For patients with “certainly relevant” positive patch test reactions, the product type responsible for the allergic contact dermatitis are shown in Table S2. In the majority of patients (15/21, 71.4%), the responsible product types were rinse‐off products such as soap and shampoo, followed by leave‐on products such as cosmetics and creams (9/21, 42.9%). Other product types included cleaning agents (detergents), deodorants, and perfumes.

**Table 3:** Clinical relevance for each positive reaction to hydroperoxides of limonene and hydroperoxides of linalool, presented for different groups of patients; patients with positive reactions to hydroperoxides of limonene or hydroperoxides of linalool, but not to any other fragrance; patients with positive reactions to both hydroperoxides of limonene and hydroperoxides of linalool, but not to any other fragrance; patients with positive reactions to hydroperoxides of limonene or hydroperoxides of linalool, and to ≥1 other fragrances; and patients with positive reactions to both hydroperoxides of limonene and hydroperoxides of linalool, and to ≥1 other fragrances

			Relevance, *n* (%)			
Reaction profile		N(total)	Unlikely/not	Possible	Probable	Certain
Only hydroperoxides of limonene	Limonene	26	5 (19.2)	14 (53.8)	4 (15.4)	3 (11.5)
Only hydroperoxides of linalool	Linalool	42	7 (16.7)	19 (45.2)	11 (26.2)	5 (11.9)
Both hydroperoxides of limonene and hydroperoxides of linalool	Limonene	16	1 (6.3)	6 (37.5)	4 (25.0)	5 (31.3)
	Linalool	16	0	6 (37.5)	4 (25.0)	6 (37.5)
Hydroperoxides of limonene + other fragrance	Limonene	13	3 (23.1)	7 (53.8)	3 (23.1)	0
Hydroperoxides of linalool + other fragrance	Linalool	16	1 (6.3)	8 (50.0)	5 (31.3)	2 (12.5)
Both hydroperoxides of limonene and hydroperoxides of linalool + other fragrance	Limonene	22	3 (13.6)	7 (31.8)	6 (27.3)	6 (27.3)
	Linalool	22	3 (13.6)	7 (31.8)	6 (27.3)	6 (27.3)
Total hydroperoxides of limonene	Limonene	77	12 (15.6)	34 (44.2)	17 (22.1)	14 (18.2)
Total hydroperoxides of linalool	Linalool	96	11 (11.5)	40 (41.7)	26 (27.1)	19 (19.8)

### 3.4 Patient characteristics

The patient characteristics according to the MOAHLFA index are shown in Table 4 for each of the defined groups (Figure 1). When group A (positive reactions to hydroperoxides of limonene and/or hydroperoxides of linalool) was compared with group F (no positive reactions to any fragrance), patients in group A were significantly less often male (24.4% vs 39.3%, *P* = 0.001), significantly older (age >40 years; 67.4% vs 53.1%, *P* = 0.002), suffer(ed) significantly less from atopic dermatitis (40.0% vs 50.7%, *P* = 0.028), and less often had the face as the primary site of dermatitis (17.0% vs 21.8%, *P* = 0.24). No notable differences were observed between patients in groups B, C, and D, except that patients in group D were almost exclusively female (10.5% males).

**Table 4:** The MOAHLFA index for the different subgroups of patients, as shown in Figure 1

	Group A; positive for hydroperoxides of linalool and/or hydroperoxides of limonene (*n* = 135)	Group B; positive for hydroperoxides of limonene (*n* = 77)	Group C; positive for hydroperoxides of linalool (*n* = 96)	Group D; positive for both hydroperoxides of limonene and hydroperoxides of linalool (*n* = 38)	Group E; positive for another fragrance (*n* = 98)	Group F; not positive for a fragrance (*n* = 588)
M	24.4	18.2	24.0	10.5	31.6	39.3
O	23.0	22.1	19.8	13.2	21.4	20.2
A	40.0	37.7	40.6	39.5	54.1	50.7
H	37.0	37.7	37.5	36.8	41.8	37.4
L	1.5	1.3	2.1	2.6	0	2.6
F	17.0	18.2	13.5	13.2	24.5	21.8
A	67.4	62.3	71.9	68.4	50.0	53.1

Abbreviations: M, male; O, occupational dermatitis; A, a (history of) atopic dermatitis; H, hand as the primary site of dermatitis; L, leg as the primary site of dermatitis; F, face as the primary site of dermatitis; A, age >40 years.

A regression analysis was performed for each of these groups (except for group F, all members of which were not allergic to a fragrance); the results are shown in Table 5. Data from group A showed that being female was a significant risk factor for contact allergy to hydroperoxides of limonene and/or hydroperoxides of linalool (odds ratio [OR] 1.91, 95%CI: 1.25‐2.91). In group A, another significant association was found for patients aged ≥40 years (OR 1.86, 95%CI: 1.26‐2.75). A decreased risk was found in patients with a current or past history of atopic dermatitis (OR 0.64, 95%CI: 0.44‐0.93). In the multivariable analysis, atopic dermatitis was no longer a significant risk factor (OR 0.71, 95%CI: 0.48‐1.06), whereas female sex became an even larger risk factor (OR 2.05, 95%CI: 1.33‐3.13). In group A, females had significantly more atopic dermatitis than males (52.2% vs 43.8%, *P* = 0.019), which might explain this finding. When group B (positive reactions to hydroperoxides of limonene) was compared with group C (positive reactions to hydroperoxides of linalool), the main difference was that age ≥40 years was a risk factor for patients in group C (OR 2.28, 95%CI: 1.43‐3.65), but not significantly so in group B (OR 1.39, 95%CI: 0.86‐2.26). Conversely, although female sex was a significant risk factor in both groups, it was a stronger risk factor in group B (OR 2.73) than in group C (OR 1.91). In the multivariable analysis, atopic dermatitis was significantly associated with being in group B (OR 0.59, 95%CI: 0.36‐0.95), suggesting that the importance of atopic dermatitis in group A was mainly driven by the subset of patients allergic to hydroperoxides of limonene, whereas a larger proportion of the subset of patients allergic to hydroperoxides of linalool were aged ≥40 years. Occupational dermatitis and primary site of dermatitis were not significant risk factors for any of the groups.

**Table 5:** Univariable and multivariable regression analysis performed for each group of patients as shown in Figure 1, with the exception of group F, with the following variables: sex, age (<40 years vs ≥40 years), (a history of) atopic dermatitis, occupational dermatitis, and primary site of dermatitis (generalized, trunk, hand, leg, face, and other)

		Group A; positive for hydroperoxides of limonene and/or hydroperoxides of linalool (*n* = 135)		Group B; positive for hydroperoxides of limonene (*n* = 77)		Group C; positive for hydroperoxides of linalool (*n* = 96)		Group D; positive for both hydroperoxides of limonene and hydroperoxides of linalool (*n* = 38)		Group E; positive for another fragrance (*n* = 98)	
Univariable regression		OR	95%CI	OR	95%CI	OR	95%CI	OR	95%CI	OR	95%CI
Sex	Male	1.00 (ref.)		1.00 (ref.)		1.00 (ref.)		1.00 (ref.)		1.00 (ref.)	
	Female	**1.91**	**1.25‐2.91**	**2.73**	**1.50‐4.97**	**1.91**	**1.17‐3.12**	**5.03**	**1.77‐14.31**	1.24	0.79‐1.95
Age (years)	<40	1.00 (ref.)		1.00 (ref.)		1.00 (ref.)		1.00 (ref.)		1.00 (ref.)	
	≥40	**1.86**	**1.26‐2.75**	1.39	0.86‐2.26	**2.28**	**1.43‐3.65**	1.82	0.90‐3.65	0.79	0.52‐1.21
Atopic dermatitis	No	1.00 (ref.)		1.00 (ref.)		1.00 (ref).		1.00 (ref.)		1.00 (ref.)	
	Yes	**0.64**	**0.44‐0.93**	0.63	0.37‐1.02	0.67	0.44‐1.03	0.66	0.34‐1.28	1.24	0.81‐1.90
Occupational dermatitis	No	1.00 (ref.)		1.00 (ref.)		1.00 (ref.)		1.00 (ref.)		1.00 (ref.)	
	Yes	1.16	0.79‐1.81	1.09	0.62‐1.91	0.93	0.55‐1.59	0.56	0.22‐1.47	1.04	0.62‐1.74
Site of dermatitis	Generalized	1.00 (ref.)		1.00 (ref.)		1.00 (ref.)		1.00 (ref.)		1.00 (ref.)	
	Trunk	1.84	0.61‐5.5	2.29	0.37‐14.35	1.56	0.50‐3.26	1.49	0.20‐11.00	0.61	0.18‐2.14
	Hand	1.45	0.63–‐3.37	2.87	0.67‐12.38	0.99	0.50‐4.84	1.37	0.30‐6.18	0.86	0.39‐1.88
	Leg	1.01	0.19‐5.38	1.81	0.15‐21.29	1.01	0.19‐5.38	1.81	0.15‐21.30	NA	NA
	Face	1.15	0.47‐2.82	2.72	0.60‐12.54	0.61	0.23‐1.60)	0.85	0.16‐4.52	0.90	0.39‐2.06
	Other	1.98	0.84‐4.65)	4.30	0.99‐18.58)	1.56	0.53‐3.03	2.00	0.44‐9.05	0.58	0.25‐1.34
**Multivariable regression (variables entered when *P* < 0.1 in univariable regression)**											
Sex	Male	1.00 (ref.)		1.00 (ref.)		1.00 (ref.)		1.00 (ref.)			
	Female	**2.05**	**1.33‐3.13**	**2.89**	**1.58‐5.26**	**2.04**	**1.24‐3.35**	**5.21**	**1.83‐14.87**		
Age (years)	<40	1.00 (ref.)				1.00 (ref.)		1.00 (ref.)			
	≥40	**1.73**	**1.14‐2.62**			**2.21**	**1.35‐3.62**	1.94	0.96‐3.91		
Atopic dermatitis	No	1.00 (ref.)		1.00 (ref.)		1.00 (ref.)					
	Yes	0.71	0.48‐1.06	**0.58**	**0.36‐0.94**	0.81	0.51‐1.29				

Abbreviations: CI, confidence interval; NA, not applicable; OR, odds ratio.

Variables were entered into the multivariable regression analysis if the *P*‐value was <0.1 in the univariable regression analysis. Values in bold are statistically significant (p < 0.05).

### 3.5 Concomitant contact allergies

The proportions of patients with contact allergies other than to either hydroperoxides of limonene or hydroperoxides of linalool were 75.6% (95%CI: 68.4%‐82.8%) for group A (patients allergic to hydroperoxides of limonene and/or hydroperoxides of linalool), and 84.2% (95%CI: 72.6%‐95.8%) for group D (allergic to both hydroperoxides) (Table 6). The proportion of patients with additional contact allergies but not to fragrances and/or colophonium was 72.6% (95%CI: 65.1%‐80.1%) for group A.

**Table 6:** The proportion of patients with at least 1 additional contact allergy other than to hydroperoxides of limonene and hydroperoxides of linalool, the proportion of patients with at least 1 additional non‐fragrance allergy, the median additional (non‐fragrance) reactions in group A (patients allergic to hydroperoxides of limonene and/or hydroperoxides of linalool (Figure 1), and the subsets of patients allergic to only hydroperoxides of limonene, patients allergic to only to hydroperoxides of linalool, and patients allergic to both hydroperoxides of limonene and hydroperoxides of linalool

		Proportion with ≥ 1 additional contact allergies (other than to hydroperoxides)	Proportion with ≥ 1 additional contact allergies (other than to hydroperoxides or fragrance/colophonium)	Additional reactions	Additional reactions (other than to fragrance/colophonium)
	Total (*N*)	*n* (%)	*n* (%)	Median (IQR)	Median (IQR)
Group A (hydroperoxides of limonene and/or hydroperoxides of linalool)	135	102 (75.6)	98 (72.6)	2 (1‐6)	1 (0‐4)
Only hydroperoxides of limonene	39	28 (73.7)	27 (70.7)	1 (0‐7)	1 (0‐5)
Only hydroperoxides of linalool	58	42 (72.4)	41 (70.7)	1 (0‐5)	1 (0‐3.25)
Both hydroperoxides of limonene and hydroperoxides of linalool	38	32 (84.2)	30 (78.9)	3.5 (1‐9)	2 (1‐5)

IQR, interquartile range.

Including both hydroperoxides of limonene and hydroperoxides of linalool, a total of 233 patients (28.4%, 95%CI: 25.3%‐31.4%) had at least 1 positive reaction to a fragrance. Of these 233 patients, 98 (11.9%, 9.7%‐14.1%) had a positive reaction to a fragrance but not to either hydroperoxides of limonene or hydroperoxides of linalool, 53 patients (6.5%, 95%CI: 4.8%‐8.2%) reacted to both at least 1 fragrance and hydroperoxides of limonene and/or hydroperoxides of linalool, and the remaining 82 patients (10.0%, 95%CI: 7.9%‐12.0%) reacted only to hydroperoxides of limonene and/or hydroperoxides of linalool, but not to any other fragrance. Table 7 shows the numbers of concomitant fragrance reactions per patch tested fragrance. An important observation is that, regarding patients with contact allergy to hydroperoxides of limonene and/or hydroperoxides of linalool, a significantly larger proportion had a concomitant contact allergy to a fragrance and/or colophonium (39.3%, 95%CI: 31.3%‐47.5%) than patients who were not allergic to hydroperoxides of limonene and/or hydroperoxides of linalool (14.3%, 95%CI: 11.7%‐16.9%). On analysis of group A, patients who were allergic to both hydroperoxides of limonene and hydroperoxides of linalool more frequently reacted positively to additional fragrance allergens and/or colophonium (63.2%, 95%CI: 47.9%‐78.5%) than patients who were allergic to hydroperoxides of limonene (33.3%, 95%CI: 18.5%‐48.1%) and significantly more frequently than patients who were allergic to hydroperoxides of linalool alone (27.6%, 95%CI: 16.1%‐39.1%).

**Table 7:** The number of patients with positive reactions to each of the fragrance (markers) allergens and/or colophonium, presented for patients allergic to hydroperoxides of limonene and/or hydroperoxides of linalool, for the subsets of patients allergic to only hydroperoxides of limonene, patients allergic to only to hydroperoxides of linalool, and patients allergic to both hydroperoxides of limonene and hydroperoxides of linalool, and for all other patients not allergic to either hydroperoxides of limonene or hydroperoxides of linalool

Fragrance	Allergic to hydroperoxides of limonene and/or hydroperoxides of linalool (*N* = 135), *n* (%)	Allergic only to hydroperoxide of limonene (*N* = 39), *n* (%)	Allergic only to hydroperoxide of linalool (*N* = 58), *n* (%)	Allergic to both hydroperoxides of limonene and hydroperoxides of linalool (*N* = 38), *n* (%)	All other patients not allergic to either hydroperoxides of limonene or hydroperoxides of linalool (*N* = 686), *n* (%)
European baseline series					
Fragrance mix I^a^	15 (11.1)	3 (7.7)	7 (12.1)	5 (13.2)	16 (2.3)
Fragrance mix II^b^	24 (17.8)	3 (7.7)	8 (13.8)	13 (34.2)	32 (4.7)
Hydroxyisohexyl 3‐cyclohexenecarboxyaldehyde (Lyral)^b^	10 (7.4)	1 (2.6)	3 (5.2)	6 (15.8)	29 (4.2)
*Myroxylon pereirae* (balsam of Peru)	10 (7.4)	3 (7.7)	3 (5.2)	4 (10.5)	13 (1.9)
Colophonium (rosin)	4 (3.0)	1 (2.6)	0	3 (7.9)	21 (3.1)
Subtotal (≥1 of the above)	39 (28.9)	9 (23.1)	14 (24.1)	16 (42.1)	72 (10.5)
Fragrance series					
Amyl cinnamyl alcohol^a^	2 (1.5)	0	0	2 (5.3)	2 (0.3)
Anisyl alcohol (anise alcohol)	0	0	0	0	0
Benzyl alcohol	0	0	0	0	3 (0.4)
Benzyl benzoate	0	0	0	0	0
Benzyl cinnamate	0	0	0	0	0
Benzyl salicylate	1 (0.7)	0	1 (1.7)	0	0
Cinnamic alcohol	3 (2.2)	1 (2.6)	0	2 (5.3)	5 (0.7)
Cinnamal^a^	6 (4.4)	1 (2.6)	1 (1.7)	4 (10.5)	10 (1.5)
Citral^b^	8 (5.9)	1 (2.6)	4 (6.9)	3 (7.9)	5 (0.7)
Citronellol^b^	4 (3.0)	1 (2.6)	2 (3.4)	1 (2.6)	3 (0.4)
Coumarin^b^	2 (1.5)	1 (2.6)	0	1 (2.6)	2 (0.3)
Farnesol^b^	4 (3.0)	0	1 (1.7)	3 (7.9)	1 (0.1)
Geraniol^a^	6 (4.4)	2 (5.1)	2 (3.4)	2 (5.3)	2 (0.3)
Hexyl cinnamal^b^	1 (0.7)	0	0	1 (2.6)	2 (0.3)
Hydroxycitronellal^a^	7 (5.2)	0	4 (6.9)	3 (7.9)	7 (1.0)
Isoeugenol^a^	8 (5.9)	3 (7.7)	2 (3.4)	3 (7.9)	13 (1.9)
Butylphenyl methylpropional (Lilial)	4 (3.0)	1 (2.6)	1 (1.7)	2 (5.3)	2 (0.3)
Methyl 2‐octynoate (methyl heptine carbonate)	1 (0.7)	0	1 (1.7)	0	1 (0.1)
α‐Isomethyl ionone (γ‐methylionone)	0	0	0	0	1 (0.1)
*Evernia prunastri* (oakmoss absolute)^a^	11 (8.1)	3 (7.7)	3 (5.2)	5 (13.2)	6 (0.9)
*Evernia furfuracea* (tree moss)	5 (3.7)	0	2 (3.4)	3 (7.9)	7 (1.0)
Amyl cinnamal (α‐amyl cinnamic aldehyde)^a^	1 (0.7)	0	0	1 (2.6)	1 (0.1)
Eugenol^a^	7 (5.2)	2 (5.1)	2 (3.4)	3 (7.9)	1 (0.1)
d‐Limonene	1 (0.7)	0	0	1 (2.6)	0
Linalool	1 (0.7)	0	0	1 (2.6)	0
Subtotal (≥1 of the above)	36 (26.7)	8 (20.5)	12 (20.7)	16 (42.1)	86 (12.5)
Total (≥1 fragrance/colophonium allergies)	53 (39.3)	13 (33.3)	16 (27.6)	24 (63.2)	98 (14.3)

a Denotes all allergens tested in fragrance mix I.

b Denotes all allergens tested in fragrance mix II.

## 4 DISCUSSION

In our cohort of consecutively patch tested dermatitis patients, 9.4% and 11.7% showed positive patch test reactions to hydroperoxides of limonene and hydroperoxides of linalool, respectively, supporting the recent proposal to include them in the EBS.^15^ This is a higher number than found in recent studies, in which prevalences of contact allergy to hydroperoxides of limonene ranged from 2.5% to 5.4%, and prevalences of contact allergy to hydroperoxides of linalool ranged from 3.9% to 7.7%.^6^, ^8^, ^9^ However, in multicentre studies, a large variation in positive patch test reactions between centres was observed; for example, in one study, prevalences of contact allergy to hydroperoxides of limonene ranged from 0% to 24.8%.^8^ A possible explanation for the large number of positive reactions to the hydroperoxides could be that our centre is a tertiary referral centre, so more patients with severe and/or persistent dermatitis are seen than in other centres. This could also explain the large proportion of additional positive reactions observed for patients with positive reactions to hydroperoxides of limonene and/or hydroperoxides of linalool, and the large number of fragrance‐positive patients overall. Concerning doubtful and irritant reactions to both hydroperoxides of limonene and hydroperoxides of linalool—in our cohort, there were many doubtful (17.2% and 21.9%, respectively) and few irritant (0.9% and 1.9%, respectively) reactions—large variations in the numbers of doubtful and irritant reactions have been observed in different studies. For instance, Deza et al observed 0.4% doubtful and 1.5% irritant reactions to hydroperoxides of limonene 0.3% pet., whereas Bennike et al observed 13.7% doubtful and 5.8% irritant reactions.^6^, ^8^ An even larger variation in the percentages of doubtful and irritant reactions has been reported for hydroperoxides of linalool. Our observation that the majority of positive reactions to either hydroperoxides of limonene or hydroperoxides of linalool were weak positive (+) is in line with the literature.^7^, ^16^, ^17^, ^18^

On the basis of our results, a low RI (<0) and a high PR (>80%) for both hydroperoxides of limonene and hydroperoxides of linalool were calculated, indicative of a problematic patch test concentration.^13^, ^14^ Considering that the low RIs for both allergens are mostly caused by a high number of doubtful reactions, and only a small number of irritant reactions, a reasonable assumption would be that patch testing with higher concentrations might improve the diagnostic performance. Differentiation between doubtful reactions and irritant reactions can be difficult, as can be deduced from the variation in doubtful and irritant reactions in previous studies. It can therefore not be excluded that, even though all visual readings were performed by an experienced dermatologist, some of the doubtful reactions were irritant. If this was the case, it might be prudent to test both allergens in a lower concentration. An additional concern regarding testing at higher concentrations is the higher risk of active sensitization, as studies have shown that an irritant effect can increase this risk.^19^ Christensson et al patch tested dermatitis patients and healthy controls with sequentially diluted concentrations of oxidized limonene and oxidized linalool; for both, an increasing concentration led to more irritant reactions, although this effect was stronger for oxidized limonene than for oxidized linalool.^20^ The highest tested concentration of oxidized linalool (20%, most likely containing 3.34% hydroperoxides of linalool, on the basis of the presence of 1.0% hydroperoxides of linalool in 6.0% oxidized linalool)^17^ showed a mean irritation score of 1.63 points, and a maximum of 4 points, based on a scoring system ranging from 0 to 9 developed by Basketter et al,^21^ in which an irritant reaction would be noted from 2 to 3 points.^20^ Studies have been performed with lower patch test concentrations of hydroperoxides of limonene (0.1% and 0.2%) and hydroperoxides of linalool (0.25% and 0.5%), and have concluded that the current patch test concentrations are preferred over lower concentrations, as too many positive reactions might be missed.^8^, ^9^ The observed high PR further supports the argument that the current patch test concentrations might be too low. Future studies should be performed to investigate the ideal patch test concentrations for hydroperoxides of limonene and hydroperoxides of linalool.

Clinical relevance is generally difficult to ascertain, as it depends on how well and how diligent a patient reads product labels and identifies the presence of contact allergens in the product. For hydroperoxides of limonene and hydroperoxides of linalool, an additional limitation is that these are not mentioned as such on labels. Therefore, patients have to look for limonene and/or linalool, as these are among the 26 fragrances for which labelling is required on cosmetic and detergent products in the EU.^22^ Studies have shown that fine fragrances and essential oils, which often contain limonene and/or linalool, also contain hydroperoxides as a result of autoxidation.^23^, ^24^ In the current study, the designation “certain” clinical relevance was reserved for patients who showed a clear temporal relationship between body site‐specific exposure to a product containing limonene and/or linalool and dermatitis at that body site, even though the presence of actual hydroperoxides was not confirmed by analysis of the products, and no open use test or repeated open application test was performed. Notwithstanding these limitations, almost 20% of the reactions were evaluated as being of “certain” clinical relevance, and at least another 20% were of “probable” clinical relevance.

Patients who are allergic to hydroperoxides of limonene and/or hydroperoxides of linalool were mostly female, were aged 40 years, and less frequently had (a history of) atopic dermatitis, in line with previous literature. The lower prevalence of atopic dermatitis in this patient group might explain the low number of irritant reactions to the hydroperoxides, as (a history of) atopic dermatitis is associated with increased susceptibility to irritants.^25^ A recent study, however, did not find any differences in the prevalences of atopic dermatitis between patients with positive reactions to hydroperoxides of limonene and/or hydroperoxides of linalool, and patients with irritant reactions.^9^ A strength of the current investigation is that D7 readings were performed, as, without this late reading, approximately 8% and 4% of positive reactions to hydroperoxides of limonene and hydroperoxides of linalool, respectively, would have been missed.

In conclusion, high prevalences of contact allergy to hydroperoxides of limonene and hydroperoxides of linalool have once again been observed, supporting the proposed inclusion in the EBS. Furthermore, 40% of all reactions to hydroperoxides of limonene and hydroperoxides of linalool were of either “probable” or “certain” clinical relevance. Although varying proportions of doubtful and irritant reactions have been reported in the literature, the low RIs and high PRs calculated for both hydroperoxides of limonene and hydroperoxides of linalool indicate that the ideal patch test concentration might be higher than the currently tested concentrations, although the risk of active sensitization must be kept in mind. A large number of patients who are allergic to hydroperoxides of limonene and/or hydroperoxides of linalool have additional contact allergies, both to fragrances and to other non‐fragrance contact allergens.

## CONFLICT OF INTEREST

The authors declare no potential conflict of interests.

## Supporting information


**Table S1** Overview of all fragrance allergens (and colophonium) patch tested in each consecutive patient at our patch test clinic. Fragrance mix I, Myroxylon pereirae and colophonium are tested with TRUE Test; all other fragrance allergens are tested in petrolatum in Van der Bend chambers.
**Table S2** The product category that caused allergic contact dermatitis in subjects with a contact allergy of certain clinical relevance to hydroperoxides of limonene and/or hydroperoxides of linalool. The presence of limonene and/or linalool in the product was ascertained by the patients themselves, and by the dermatologist if a patient brought the suspected products to the outpatient clinic.
