# Supplementary material for: Contact sensitization to hydroperoxides of limonene and linalool: Results of consecutive patch testing and clinical relevance
Source: Contact Dermatitis. 2018 Oct 31;80(2):101–9. doi: 10.1111/cod.13137 (PMC6587870; doi:10.1111/cod.13137)
Supplement: Supplementary file 1 — Table S1 Overview of all fragrance allergens (and colophonium) patch tested in each consecutive patient at our patch test clinic. Fragrance mix I, Myroxylon pereirae and colophonium are tested with TRUE Test; all other fragrance allergens are tested in petrolatum in Van der Bend chambers. Table S2 The product category that caused allergic contact dermatitis in subjects with a contact allergy of certain clinical relevance to hydroperoxides of limonene and/or hydroperoxides of linalool. The presence of limonene and/or linalool in the product was ascertained by the patients themselves, and by the dermatologist if a patient brought the suspected products to the outpatient clinic. [file COD-80-101-s001.docx]

**Table S1**: Overview of all fragrance allergens (and colophonium) patch tested in each consecutive patient at our patch test clinic. Fragrance mix I, myroxylon pereirae, and colophonium are tested with the TRUE Test®, all other fragrance allergens are tested in petrolatum in Van der Bend chambers.

| **Fragrance** | **Concentration and vehicle** |
| --- | --- |
| ***European Baseline series*** |  |
| Fragrance mix I | 430 μg/cm² (TRUE Test®) |
| Fragrance mix II | 14.0% pet. |
| Hydroxyisohexyl 3-cyclohexenecarboxyaldehyde (Lyral) | 5.0% pet. |
| Myroxylon Pereirae (Balsam of peru) | 800 μg/cm² (TRUE Test®) |
| Colophonium (resin) | 850 μg/cm² (TRUE Test®) |
| ***Fragrance series*** |  |
| Amyl cinnamyl alcohol | 5.0% pet. |
| Anisyl alcohol (Anise Alcohol) | 1.0% pet. |
| Benzyl alcohol | 10.0% pet. |
| Benzyl benzoate | 1.0% pet |
| Benzyl cinnamate | 5.0% pet. |
| Benzyl salicylate | 1.0% pet. |
| Cinnamic alcohol | 2.0% pet |
| Cinnamic aldehyde | 1.0% pet. |
| Citral | 2.0% pet |
| Citronellol | 1.0% pet. |
| Coumarin | 5.0% pet. |
| Farnesol | 5.0% pet. |
| Geraniol | 2.0% pet |
| Hexyl cinnamic aldehyde (Hexyl Cinnamal) | 10.0% |
| Hydroxycitronellal | 2.0% pet |
| Isoeugenol | 2.0% pet |
| Butylphenyl Methylpropional (Lilial) | 10.0% pet. |
| D-Limonene | 2.0% pet |
| Linalool | 10.0% pet. |
| Methyl 2-Octynoate (Methyl heptene carbonate) | 1.0% pet. |
| Alpha-isomethyl Ionone (gamma-Methylionone) | 1.0% pet. |
| Evernia Prunastri (Oakmoss absolute) | 2.0% pet |
| Evernia Furfuracea (Treemoss) | 1.0% pet. |
| Amyl Cinnamal (alpha amyl Cinnamic aldehyde) | 1.0% pet. |
| Eugenol | 2.0% pet |
| Hydroperoxides of Linalool | 1.0% pet. |
| Hydroperoxides of Limonene | 0.3% pet. |

| **subject** | **Contact allergy to hydroperoxides of:** | ***Product category*** | | | | |
| --- | --- | --- | --- | --- | --- | --- |
|  |  | **soap, shampoo (rinse off products)** | **cosmetic, creams (stay-on products)** | **cleaning agents (detergents)** | **deodorant** | **perfumes** |
| 1 | limonene | x |  |  |  |  |
| 2 | limonene | x |  |  |  |  |
| 3 | limonene | x | x |  |  |  |
| 4 | limonene | x |  |  |  | x |
| 5 | limonene |  | x |  |  |  |
| 6 | linalool | x |  |  |  |  |
| 7 | linalool | x |  |  |  |  |
| 8 | linalool |  | x |  |  |  |
| 9 | linalool |  | x |  |  |  |
| 10 | linalool |  | x |  |  |  |
| 11 | both |  | x | x |  |  |
| 12 | both | x |  | x |  |  |
| 13 | both | x |  |  |  |  |
| 14 | both | x | x |  | x | x |
| 15 | both | x |  |  |  |  |
| 16 | both | x |  |  | x | x |
| 17 | both | x |  |  | x |  |
| 18 | both | x | x | x |  | x |
| 19 | both |  |  |  | x |  |
| 20 | both | x |  |  | x |  |
| 21 | both | x | x |  |  |  |

**Table S2:** The product category which caused allergic contact dermatitis in subjects with a contact allergy of certain clinical relevance to either/both hydroperoxides of limonene and/or linalool. Presence of either or both limonene and/or linalool in the product was ascertained by the patients themselves, and by the dermatologist in case a patient brought their suspected products to the outpatient clinic.
